# How Do We Assess Patient Skills in a Competence-Based Program? Assessment of Patient Competences Using the Spanish Version of the Prolapse and Incontinence Knowledge Questionnaire and Real Practical Cases in Women with Pelvic Floor Disorders

**DOI:** 10.3390/ijerph18052377

**Published:** 2021-03-01

**Authors:** Beatriz Sánchez-Sánchez, Beatriz Arranz-Martín, Beatriz Navarro-Brazález, Fernando Vergara-Pérez, Javier Bailón-Cerezo, María Torres-Lacomba

**Affiliations:** 1 Physiotherapy in Women’s Health Research Group, Physiotherapy Department, Faculty of Medicine and Health Sciences, University of Alcalá, Alcalá de Henares, 28871 Madrid, Spain; beatriz.sanchez@uah.es (B.S.-S.); beatriz.arranz@edu.uah.es (B.A.-M.); fernando.vergara@uah.es (F.V.-P.); bailonfisioterapia@gmail.com (J.B.-C.); maria.torres@uah.es (M.T.-L.); 2 Department of Physiotherapy, Centro Superior de Estudios Universitarios La Salle, Universidad Autónoma de Madrid, 28023 Madrid, Spain

**Keywords:** validation, Spanish, prolapse and incontinence knowledge questionnaire (PIKQ), patient education, patient competence, patient competence assessment, competence assessment tools, pelvic floor disorders

## Abstract

Therapeutic patient education programs must assess the competences that patients achieve. Evaluation in the pedagogical domain ensures that learning has taken place among patients. The Prolapse and Incontinence Knowledge Questionnaire (PIKQ) is a tool for assessing patient knowledge about urinary (UI) and pelvic organ prolapse (POP) conditions. The aim of this study was to translate the Prolapse and Incontinence Knowledge Questionnaire (PIKQ) into Spanish and test its measurement properties, as well as propose real practical cases as a competence assessment tool. The cross-cultural adaptation was conducted by a standardized translation/back-translation method. Measurement properties analysis was performed by assessing the validity, reliability, responsiveness, and interpretability. A total of 275 women were recruited. The discriminant validity showed statistically significant differences in the PIKQ scores between patients and expert groups. Cronbach’s alpha revealed good internal consistency. The test–retest reliability showed excellent correlation with UI and POP scales. Regarding responsiveness, the effect size, and standardized response mean demonstrated excellent values. No floor or ceiling effects were shown. In addition, three “real practical cases” evaluating skills in identifying and analyzing, decision making, and problem-solving were developed and tested. The Spanish PIKQ is a comprehensible, valid, reliable, and responsive tool for the Spanish population. Real practical cases are useful competence assessment tools that are well accepted by women with pelvic floor disorders (PFD), improving their understanding and their decision-making regarding PFD.

## 1. Introduction

Female pelvic floor disorders (PFD) include a wide variety of clinical conditions, among which urinary incontinence (UI) and pelvic organ prolapse (POP) stand out [1]. Epidemiological studies show that about one quarter of women report at least one PFD, the main types being UI and POP [2,3,4]. Although PFDs do not pose a risk to women’s lives, they significantly reduce women’s quality of life (QoL) and impact the social, domestic, sexual, physical, psychological, or occupational aspects of their lives [5,6]. Even if women recognize their PFD symptoms as a significant problem, many do not seek medical attention for several reasons. Some of them accept their situation as a normal process of aging and childbirth, believing that there is no treatment for their condition. Shame or modesty can also affect this decision. The lack of information from health professionals given to women and women’s lack of knowledge about what PFDs and their treatments favor the fact that they do not seek professional treatment [7,8]. In fact, a Gavira et al. study indicated that although 50% of women indicated that UI affects their QoL, 77% of them did not seek any aid [9]. The study by L. Wischnitzer showed that knowledge of UI is important because it encourages women to request timely treatment [10].

PFD treatment can be surgical or conservative in symptomatic women, and preventive in asymptomatic women. Much of the success of these treatments is based on the women’s adherence. Patient education is associated with good rates of adherence [11]. For a suitable level of adherence, women should know what PFDs are, how to prevent them, and their risks and treatments. They must also master the skills related to the analysis and interpretation of clinical signs and scenarios; problem-solving; making informed decisions; and the ability to discuss specific health goals and challenges, to describe their symptoms and complaints, and to participate in the development of their own health plans together with health professionals, etc. [12,13]. However, there is a paucity of detailed papers on the evaluation of the patient competence in PFD educational programs [13]. Some studies have reported that educational interventions on PFD improve symptoms and QoL in women with PFD [14,15].

Women’s PFD knowledge varies by study population [16,17,18]. Evaluation in the pedagogical domain ensures that learning has taken place among patients. It also allows us to know women’s initial knowledge and beliefs, as well as how women’s knowledge is constructed and organized, as well as the confidence that they have in their knowledge, etc. This is added to other competence assessment tools to evaluate skills relating to identification, analysis, and interpretation; problem-solving; and making informed decisions, which verify that health behavior transformations take place in patients. In this sense, the Prolapse and Incontinence Knowledge Questionnaire (PIKQ) is a validated questionnaire that assesses women’s knowledge about UI and POP [19]. Specifically, this test measures the epidemiology, pathogenesis, diagnosis and treatment knowledge surrounding these clinical conditions. The original language of PIKQ is English, so a cultural adaptation and validation for use in Spain are necessary in order to have a valid tool that allows us to assess knowledge in the Spanish population. In addition, “real practical cases” were proposed and tested as a competence assessment tool in which women actively participate.

## 2. Materials and Methods

A cross-sectional observational study was carried out at the Physiotherapy in Women’s Health Research Unit of the Alcalá University (Madrid, Spain), from March 2017 to February 2020. This study was approved by the Príncipe de Asturias Hospital’s Clinical Research Ethics Committee in Alcalá de Henares (Madrid, Spain). The study was conducted in accordance with the Declaration of Helsinki. The validation study was conducted in two phases. In the first phase, the PIKQ questionnaire was translated and culturally adapted to Spanish, and in the second phase, its measurement properties were analyzed. 

### 2.1. Translation and Cultural Adaptation

The cross-cultural adaptation of the PIKQ was conducted in three phases pursuant to the International Society for Pharmacoeconomics and Outcomes Research (ISPOR) Task Force for Translation and Cultural Adaptation [20]. In the first phase, the questionnaire was translated and culturally adapted for two English–Spanish translators (native Spanish speakers) who worked separately and obtained two versions in Spanish that proved to be equivalent to the PIKQ original version. The translators proofread the translations and, finally, with the research team, who are experts in PFD, agreed to the synthesis of the Spanish translation. 

In the second phase, two Spanish–English translators (native English speakers) worked autonomously with the Spanish translation to create two English versions. After this, an expert committee, who are specialized in PFD and psychometric methods, with these two back-translations and the Spanish-translated version, agreed on the prefinal Spanish version of the PIKQ and on its grammatical and semantic equivalence to the original.

Cognitive interviews were performed in a third phase, to test the comprehensibility. The prefinal Spanish version of PIKQ was provided to 20 native Spanish-speaking women who fulfilled the inclusion criteria (patient or expert group criteria). After filling in the questionnaire, the women were interviewed to determine if they could understand all questions, and the difficulties were identified and corrected. Finally, the final Spanish version of PIKQ was obtained. The study reporting followed the “Strengthening the Reporting of Observational studies in Epidemiology” (STROBE) guidelines.

### 2.2. Testing Measurement Properties

#### 2.2.1. Participants and Procedure

For the patient group, 147 consecutive women with PFD symptoms who fulfilled the inclusion criteria were recruited at the Women’s Health Research Unit of the University of Alcalá and assessed by physiotherapists of the Physiotherapy in Women’s Health Research Group. These women were informed about the study and were invited to participate, and written informed consent was obtained from all women. The inclusion criteria for the patient group were: female with PFD symptoms, over 18 years of age, being able to read and to understand the Spanish language. For PFD diagnosis, women were subjected to a clinical assessment, which included a physical examination and measured urinary and POP symptoms (symptoms of stress and urinary urgency and symptoms of bulging or feeling something coming down or out of the vagina). Women were excluded if they were employed or students in a medical field, were currently pregnant, or did not have the mental capacity to fill in the questionnaire. For the control group, 128 women were recruited from the 4th year physiotherapy students and professors at Alcalá University and physiotherapists experts in women’s health. The inclusion criteria were: above 18 years of age, being able to read and to understand the Spanish language. Sample size was determined according to the general recommendations of Terwee et al. [21], who recommend a subject-to-item ratio of at least 4:1, with a minimum of 100 subjects. 

**Figure 1:**
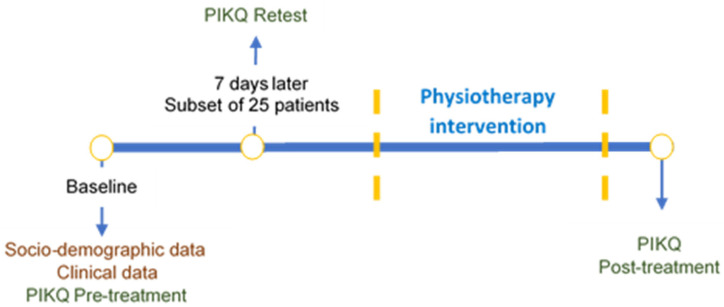
Flowchart of Prolapse and Incontinence Knowledge Questionnaire (PIKQ) administration in patient group.

At baseline, all women (from patients’ and experts’ group) completed the Spanish PIKQ, and their sociodemographic and clinical data were collected. One week later, from the patient group, a subsample of 25 women filled in the PIKQ again, in order to analyze the test–retest reliability. No treatment was delivered during this time; this interval was chosen to ensure that women’s knowledge remained unchanged and long enough to ensure that they would not recall their baseline responses. Women from the patient group, after performing a pelvic physiotherapy treatment that included a pelvic health educational strategy, were supplied again with the PIKQ to evaluate responsiveness (Figure 1). The average time was recorded.

#### 2.2.2. Demographic Data and PIKQ Score Determination

The tools used in this study included a participant characteristics form and the Spanish version of the PIKQ. 

Participant Characteristics

The women’s baseline characteristics collected were: age, parity, educational level, occupational status, income, marital status, vaginal delivery, cesarean, menopause, and UI or POP symptoms.

Prolapse and Incontinence Knowledge Questionnaire 

The PIKQ is a self-administered questionnaire developed by Aparna et al. to assess levels of patient knowledge about UI and POP. It contains 24 items, divided into two sections: UI and POP sections. Each section has 12 items, to assess the knowledge about the etiology, diagnosis and treatment of UI and POP. The score range for each item was 0 (incorrect or unknown answer) to 1 (correct answer). Total UI and POP scale scores are computed by summing the number of correct responses within each scale. In each scale, the minimum score is 0 and the maximum is 12. 

#### 2.2.3. Data Analysis

Statistical analyses were conducted using SPSS Version 23.0 (IBM Corp). Descriptive statistics were calculated using the arithmetic mean and standard deviation (SD) as indices of central tendency and dispersion for the quantitative variables or using the median and interquartile ranges when wide dispersions conditioned the interpretation of the variable. Absolute and relative percentage frequencies were used for the categorical variables. The inferential analysis was estimated with a 95% confidence interval (CI), considering a *p*-value <0.05 as statistically significant.

The Consensus-Based Standards for the Selection of Health Measurement Instruments recommendations (COSMIN) were followed to evaluate the validity, reliability, responsiveness, interpretability and feasibility of the Spanish final version of PIKQ.

**Validity**

Validity was evaluated in terms of content and discriminative construct validity. Although content validity for assessing the ability of items to collect knowledge status was guaranteed during the development of the original PIKQ, the face/content validity of the Spanish version was assessed by the expert committee, who assessed the ability of items to assess all dimensions, as well as the pilot study women’s opinions.

A predefined hypothesis was established regarding discriminative construct validity (or known-groups validity). Statistically significantly higher scores were expected in the expert group (women employed or students in a medical field) compared to the patient group, for both PIKQ-IU and PIKQ-POP sections. A large effect size for mean differences was expected. Student’s T test was employed for this comparison and Cohen’s d corrected for sample sizes of the groups for effect size calculation.

**Reliability**

Reliability was assessed by internal consistency and the test–retest reliability. The internal consistency is the degree of interrelatedness among all the items in each section of the questionnaire. It was measured by means of Cronbach’s alpha (α), considering a value of ≥0.7 as acceptable [22]. The higher the value, the greater the internal consistency [21,23]. 

The test–retest reliability is the degree to which a measurement is free from error [24]. The test–retest reliability for the total scores was analyzed with two-way random effects, single measures, and an absolute agreement intraclass correlation coefficient (ICC) model assuming a value ≥0.7 as acceptable [21,22]. Derived from the test–retest reliability study, the standard error of measurement (SEM) and smallest detectable change (SDC) were calculated. The SEM was calculated following the formula $SD\times \sqrt{1-ICC}$, where SD is the standard deviation of the mean of all observed scores, and the ICC is the reliability estimator. The smallest detectable change (SDC) was calculated as $SEM\times 1.96\times \sqrt{2}$ at an individual level and as $SEM\times 1.96\times \sqrt{2}/n$ at a group level [25].

**Responsiveness**

Responsiveness is the sensitivity of PIKQ to detect significant changes. It was assessed in the patient group, who underwent a pelvic physiotherapy treatment that included an educational strategy. A comparison between the baseline and post-intervention scores of PIKQ was calculated. Based on the effects found for educative interventions in patients [13,26,27,28], it was expected that we would find good to excellent effect sizes when comparing the baseline score in PIKQ-IU and PIKQ-POP with post-treatment scores in the same questionnaires. Effect size (ES) (mean change score/SD baseline) and standardized response mean (SRM) (mean change score/SD change score) were calculated [22]. A value of 0.2–0.49 was considered small, 0.5–0.79 moderate, 0.8–0.99 good, and more than 1.0 excellent [21,23,24]. 

**Interpretability and feasibility**

The percentage of unanswered individual items and ceiling and floor effects were studied among the PIKQ sections. Ceiling or floor effects are given when more than 15% of the responses get the best or worst possible score, respectively [21]. In addition, the average administration time was calculated. 

### 2.3. Development of the Real Practical Cases

Using an iterative process, our team of pelvic floor physiotherapists, together with an educational evaluation and assessment specialist, developed three cases extracted from real contexts. These were related to the competences to be assessed (see Table 1), as well as to the knowledge to be mobilized during the discussion of the practical case with patients. Finally, practical cases were tested with 20 women who participated in the PFD therapeutic education program to evaluate the understanding and usefulness of real practical cases. Finally, real practical cases were tested in 60 women with PFD who participated in the pelvic health therapeutic education program to evaluate the understanding and usefulness of these practical cases.

## 3. Results

### 3.1. Translation and Cultural Adaptation

The translation and cultural adaptation of the Spanish version of PIKQ achieved good semantic, conceptual, idiomatic, and content equivalences. Minor discrepancies were discussed and resolved and the revisions of experts and the women in the pilot study guaranteed the content/face validity. Experts made several changes to some terms and phrases to ensure that the final version was more easily understood by women. For example, in the POP section’s introduction, “pelvic organ prolapse (bulging of the vagina, uterus, bladder, or rectum” was translated as “prolapso de órganos pélvicos (bulto en la vagina por el descenso de la vagina, del útero, vejiga o recto a través de la vagina)”; in item 8 of the POP section, “Heavy lifting on daily basis can lead to pelvic organ prolapse” was translated as “Levantar peso a diario puede provocar un prolapso de órganos pélvicos”. In the pilot study, women understood all the items, so the final Spanish version of PIKQ was obtained (Supplementary File S1). 

### 3.2. Measurement Properties of the Spanish PIKQ

#### 3.2.1. Characteristics of Participants, Interpretability, and Feasibility of PIKQ

The demographic and clinical data of women recruited are shown in Table 2. 

Regarding the interpretability of the scores, their distributions in the patient (baseline and post-treatment) and expert groups are shown in Table 3. All questionnaires received were complete. Twelve (8.2%) and five (3.4%) patients obtained the maximum score for the PIKQ-IU and PIKQ-POP scales, respectively. None of them obtained the minimum score. Therefore, there were no floor or ceiling effects in either of the two sections for their use in patients. Concerning feasibility, the average time for questionnaire completion was 10.4 (±2.9) min. 

#### 3.2.2. Validity

The revisions of the expert committee and the women in the pilot study guaranteed the content validity of the Spanish PIKQ. Therefore, the expert committee agreed that the PIKQ included the relevant dimensions for assessing patient knowledge about UI and POP conditions, and the interviewed women in the pilot study demonstrated that items were well understood. 

The results regarding the discriminative construct validity confirm the hypothesis concerning the differences in PIKQ-IU and PIKQ-POP scores between experts and patients (*p* < 0.001), showing large effect sizes (d = 1.94 for PIQK-IU and d = 2.35 for PIKQ-POP).

#### 3.2.3. Reliability

Cronbach’s alpha revealed good internal consistency for both scales, with a value of 0.745 (0.682–0.801) for PIKQ-IU and 0.758 (0.696–0.812) for PIKQ-POP.

For the test–retest reliability, all patients invited completed the questionnaires. The ICC showed a good test–retest validity for both questionnaires. Data derived from this analysis are presented in Table 4.

#### 3.2.4. Responsiveness

The predefined hypothesis regarding the good to excellent effect sizes of the therapeutic education program on the constructs measured by PIKQ-IU and PIKQ-POP was confirmed (Table 3).

### 3.3. Practical Cases Proposal

Descriptions of the real practical cases and the related competences are shown in Supplementary File S2. Competences, knowledge and behaviors, and achievement assessments are described. The PIKQ assesses knowledge related to the PFD, also allowing for the detection of beliefs regarding PFD, but it does not evaluate knowledge transference to real situations. In order to evaluate knowledge transference to real situations, which means being able to evaluate the competences shown in Table 1, a tool that can be used is “real practical cases” [29]. The three cases developed were presented to the PFD women before and after the therapeutic patient education program (Table 5). In the post-educational evaluation, most of the women reached the level of “gold medal/well done” in the assessment rubric of the proposed cases (Supplementary File S3). This, together with the opinions of the PFD women on the cases, turned out to be promising. Most women found the cases to be playful, interactive, and participatory. In addition, they also expressed that these cases allowed them to relate their knowledge and skills from the educational program. Moreover, as it was a face-to-face evaluation, it also allowed them to discuss their doubts with the physiotherapist.

## 4. Discussion

The PIKQ is proven to be a valid and reliable questionnaire to assess patient knowledge about UI and POP. It was developed and validated for the English language and has been adapted to the Turkish population [19,30]. The purpose of the present study was to translate it into Spanish and to test its measurement properties in terms of its validity, reliability, responsiveness, interpretability and feasibility, as well as proposing real practical cases as a critical competence assessment tool integrated within an education program. To the best of our knowledge, this is the first validation of the PIKQ in a Spanish population. This instrument will allow us to evaluate the population’s knowledge about the epidemiology, pathogenesis, diagnosis, and treatment of UI and POP in Spain, as well as the effect of educational treatment strategies on them. Therefore, this Spanish PIKQ provides the possibility of international multicenter studies and comparisons of the results of studies from different countries and cultures, aiding the exchange of information within the international scientific community and decreasing the costs and time spent on the process [31]. 

The PIKQ is a self-administrated knowledge questionnaire. The instructions to be self-administered and the structure of the questions are simple and easily understood, resulting in a very good acceptance by women, who did not need supplementary instructions in order to understand and fill in the questions independently. The three-point Likert system (agree, disagree, do not know) enables quick answering by women. The calculation of the final score is quick and very simple, implying an advantage for both research and clinical practice. 

The method used in the translation and cultural adaptation process of Spanish PIKQ was similar to that performed in the Turkish study [30], including a translation/back-translation method of the original English PIKQ to Spanish and then the evaluation of the measurement properties in a female sample. In the pilot study, some linguistic adaptations from the original version were necessary in order to strengthen the semantic and content equivalence. 

In the present Spanish PIKQ validation, the response rate was complete for all women who were administered the questionnaire; this could be due to the fact that the questionnaire was self-reported by the women in the consultation, and the fact that if a woman had any doubts when completing it, she could request help from the physiotherapist. Therefore, this method of filling in the questionnaire with the possibility of asking for help can solve patients’ doubts and increase the response rate. No floor or ceiling effects were found for any item.

The analysis of discriminant validity was measured, calculating the difference between women employed in the medical field or physiotherapy students and those who were not (expert group versus patient group). As expected, this analysis showed that PIKQ is able to discriminate between women with and without medical knowledge of women’s health, in line with studies that positively associate the health-related professions with better knowledge [32,33].

Cronbach’s alpha coefficients were 0.745 and 0.758 for the UI and POP dimensions, respectively. These values showed good internal consistency [22], in the same grade as the original version (0.825 and 0.895, respectively) and the Turkish version (0.678 and 0.756, respectively) [19,30]. This shows that when all items measure the same construct, the intercorrelations among items increase. A low Cronbach’s alpha suggests that these dimensions are testing different traits. Regarding test–retest reliability, this was evaluated after one week, as with the Turkish version, and demonstrated significant correlations, with a Pearson correlation coefficient of 0.995 for the UI section and 0.977 for the POP section, showing excellent correlation for both, in the same way as the Turkish version.

Regarding the three real practical cases, this study presents a test of the assessment tool real practical cases to evaluate the acquisition of skills related to identification, analysis, and interpretation of clinical signs and scenarios, problem-solving, and making informed decisions in PFD women. Furthermore, we include a rubric to evaluate real practical cases that integrate various competences. To the best of our knowledge, this is the first proposal found in the literature for real practical cases that includes a rubric for the evaluation of the competences of identification and analysis, decision making, problem-solving and information management. Most of the education programs found did not present tools assessing competences. Any patient therapeutic education program must carry out an assessment focused on the patient’s competences [12,29]. Authentic activities [34,35], close to the performance environment of a real-life situation, are considered as an essential aspect for the development and evaluation of competences. In our study, real practical cases for patients are a genuine learning opportunity and provide an evaluation method that has been recognized by PFD women as playful, participatory, and iterative, presenting an opportunity to integrate knowledge and skills.

The strengths of our study are the translation and cultural adaptation process following the standardized guidelines (phases pursuant by ISPOR) and the psychometric validation based on COSMIN recommendations, including the evaluation of responsiveness. Responsiveness or sensitivity to change was evaluated as a measurement property; in fact, in clinical trials, the outcomes measured that must be analyzed are validity, reliability, and responsiveness [36]. Responsiveness is the ability to detect changes that occur as a result of therapy or disease progression, and it has been suggested as one criterion to that can be chosen among the scales used to evaluate the efficacy of a therapeutic intervention [37]. In this way, responsiveness is a fundamental property that we decided should be assessed in this study. Measures of the responsiveness of an instrument have been described by different methods, but without a clear consensus about which is the best. In the present study, to assess responsiveness, we chose two common distribution-based methods: ES and SRM. Each method confirmed the excellent responsiveness of the UI and POP sections of Spanish PIKQ in women after physiotherapy treatment that included pelvic health educational interventions. These values cannot be compared with others because, to the best of our knowledge, this is the first study that evaluates responsiveness, despite this property being a determinant in the evaluation of whether educational strategies are effective. On the other hand, real practical cases have the versatility to be able to adapt to the different patients and PFDs, while the evaluation rubric is an element that allows for developing, with anticipated, transparent, and accepted criteria, learning during a therapeutic patient education program, and also allows us to obtain the outcome measures of this learning, which can be shared with the participants and professionals. These outcome measures can be useful for the improvement and modification of the therapeutic patient education program itself.

One of the most common difficulties in instrument validation studies is the analysis of criterion validity, because it is necessary to have an accepted “gold standard”. So, convergent construct validity could be evaluated by an analysis of the association with other instruments that assess similar constructs. As such, this is a limitation of the present study. Therefore, future research should examine other measurement properties of this Spanish version as the convergent validity using an instrument to evaluate the knowledge about UI and POP. In addition, our sample comprised only adult women, which could limit its use. Furthermore, future validation in diverse cultures and populations, such as adolescents or older women, are recommended. Regarding real practical cases, a limitation of their application as a competence assessment tool could be the cognitive level of the patients, although the cases could be adapted to the cognitive ability and understanding characteristics of each patient.

## 5. Conclusions

In conclusion, the Spanish PIKQ is a comprehensible, valid, reliable, feasible, and responsive-to-change tool for assessing patient knowledge about UI and POP conditions in the Spanish language, as well as the effect of educational treatment strategies on them, both in research and clinical interventions. 

“Real practical cases” are a useful competence assessment tool related to the identification, analysis and interpretation of clinical signs and scenarios, problem-solving, and making informed decisions. These competence assessment tools are well accepted by women with PFD.

However, more studies are needed to investigate and propose further competence assessment tools.

## Figures and Tables

**Table 1:** Competences and dimensions of real practical cases.

Competences	Dimensions
**Identify, analyze**	Identify and analyze the significant elements, summarize and integrate the different parts, organize the elements and the connection among them, deduce some ideas and/or results and provide some conclusions.
**Decision making**	Apply systematic methods to make decisions, compile and analyze data to take the most suitable decision, show certainty, be consistent with the solution adopted, collaborate with other on taking decisions.
**Communicate needs**	Intonation and volume, level of preparation of exposition, gestures and body language, clarity of exposure, ability to answer questions, speech clarity, structure, and sequence within the speech.
**Problem-solving**	Define the problem, identify strategies, propose solutions/hypotheses, evaluate potential solutions, implement solutions, evaluate results.
**Know how to manage**	Identify the different resources, access resources, use resources efficiently, assess the suitability of resources based on the results.

**Table 2:** Demographic and clinical characteristics of women. Values are numbers (percentages) unless stated otherwise.

	Patients Group (n = 147)	Experts Group (n = 128)
Age, years. $\bar{x}$ (SD)	38.57 (8.21)	24.18 (7.59)
Education level:		
Primary School	16 (10.9)	0 (0)
High School	49 (33.3)	0 (0)
College or University	82 (55.8)	127 (100)
Occupational Status		
Unemployed	30 (20.4)	0 (0)
Employed	115 (78.2)	28 (22)
Student	2 (1.4)	100 (78)
Incomes		
<EUR 12,000/year	12 (8.2)	28 (22)
EUR 12,000–24,000/year	52 (35.4)	-
EUR 24,000–36,000/year	35 (23.8)	100 (78)
EUR 36,000–48,000/year	29 (19.7)	-
>EUR 48,000	19 (12.9)	-
Vaginal delivery:		
None	7 (4.8)	120 (93.7)
One	92 (62.6)	5 (3.9)
Two	40 (27.2)	2 (1.5)
Three or more	8 (5.4)	1 (0.7)
Cesarean	11 (7.5)	1 (0.7)
Menopause	18 (12.2)	2 (1.5)
Urogynecological symptoms		
SUI	57 (38.7)	-
UUI	17 (11.5)	-
POP	12 (8.2)	-

$\bar{x}$ (SD): mean (standard deviation); -: not applicable; SUI: stress urinary incontinence; UUI: urge incontinence urinary; POP: pelvic organ prolapse.

**Table 3:** Scores and effect sizes of PIKQ for patient and expert groups.

		$\mathrm{Baseline}\;\mathrm{Score},\;\bar{x}\;(\mathrm{SD})$	$\mathrm{Post}-\mathrm{Treatment}\;\mathrm{Score},\;\bar{x}\;(\mathrm{SD})$	Effect Size (95%CI)	SRM (95%CI)
**Patients** **(n = 147)**	**PIKQ-IU**	8.99 (2.05)	11.24 (0.66)	1.09 (0.95–1.2)	1.16 (1.01–1.32)
	**PIKQ-POP**	7.15 (2.79)	10.38 (0.53)	1.16 (0.99–1.34)	1.15 (0.99–1.33)
**Experts** **(n = 128)**	**PIKQ-IU**	11.92 (0.27)	-	-	-
	**PIKQ-POP**	11.96 (0.23)	-	-	-

$\bar{x}$ (SD): mean (standard deviation); CI: confidence interval; SRM: standardized response mean; PIKQ-IU: Urinary Incontinence scale of Prolapse and Incontinence Knowledge Questionnaire; PIKQ-POP: Pelvic Organ Prolapse scale of Prolapse and Incontinence Knowledge Questionnaire; -: not applicable.

**Table 4:** Data derived from test–retest reliability analysis (n = 25).

	$\mathrm{Test},\;\bar{x}\;(\mathrm{SD})$	$\mathrm{Retest},\;\bar{x}\;(\mathrm{SD})$	SEM	SDC_ind_	SDC_group_	ICC (95%CI)
**PIKQ-IU**	8.56 (2.02)	8.52 (2.04)	0.15	0.42	0.017	0.995 (0.989–0.998)
**PIKQ-POP**	7.04 (2.56)	7.04 (2.42)	0.37	1.03	0.04	0.977 (0.951–0.990)

PIKQ-IU: Urinary Incontinence scale of Prolapse and Incontinence Knowledge Questionnaire; PIKQ-POP: Pelvic Organ Prolapse scale of Prolapse and Incontinence Knowledge Questionnaire; $\bar{x}$ (SD): mean (Standard Deviation); SEM: standard error of measurement; SDCind: smallest detectable change (individual level); SDCgroup: smallest detectable change (group level); ICC: intraclass correlation coefficient; CI: confidence interval.

**Table 5:** Therapeutic patient education program about pelvic floor health.

Session	Topic	Contents	Learning Tools	Activities	Assessment Tools
1	What do you know about PFD?	- Knowledge about the PFD symptoms and treatment options. - Beliefs about causes and evolution of PFD - Possible resources and self-efficacy facing a PFD	- Exploratory motivational interview - PIKQ - Real practical cases	- To fill in the PIKQ - To detect the PFD symptoms, the risk factors, and the treatment options of each real practical case	- PIKQ & real practical cases rubric are discussed - The notes of the contents in a notebook are requested and reviewed
2	Why PF is important?	- PF anatomy - Where PFM are - PFM function - What PFD are - Risk factors of PFD	- Anatomical prints - Pelvic bones and muscles anatomical model - Small ball	- To identify the boundaries and parts of the PF - To spatially locate the PFM in a picture and in themselves - To relate structures to functions, risk factors, and PFD	- A drawing of the PF anatomy is requested - A task of connecting anatomical structures with different functions is requested
3	How to contract the PFM correctly?	- Visualization of self PFM contraction - How to contract PFM - How to perform PFM exercises - Identifying her own PFD risk factors	- Ultrasound images of the PF and PFM contraction - Ultrasound biofeedback - Physiotherapist manual and auditory feedback - PFD risk factors sheet	- To understand the correct movement of the PFM - To watch and sense the correct contraction of the PFM - To contract the PFM in supine and sitting positions - To identify unwanted contraction of muscles close to the PFM - To fill in the PFD risk factors sheet at home.	- To review recordings of PFM contractions and identify the correct ones is requested - PFM contraction is assessed through vaginal palpation and transabdominal ultrasound - A diary of the PFM contractions practice is requested - The PFD risk factors sheet is discussed
4	How to relax the PFM and empty your bladder adequately? What are your voiding routines?	- Coordination between PFM and breathing - How to relax PFM - Urinary, defecatory and liquid intake patient habits	- Anatomical prints - Physiotherapist manual and auditory feedback - Motor imagery - Voiding and intake diary	- To try to contract the PFM when breathing out - To try to relax the PFM when breathing in - To fill in the voiding and intake diary during three consecutive days	- On-site test on PFM contraction and relaxation - The voiding and intake diary is requested and saved for later review
5	How important is the position of the pelvis?	- Pelvis positions during PFM exercises - Pelvis position during daily tasks - Pelvis position during micturition and defecation	- Anatomical prints - Pelvic bones and muscles anatomical model - Swiss ball	- To identify and practice the neutral pelvic position and the anterior and posterior pelvic tilt sitting and standing - To deduce which pelvic alignment assists each excretory process	- On-site test on pelvic alignment
6	What is the role of the PFM in intra-abdominal pressure?	- Coordination between PFM and abdominal muscles - Knack maneuver - How to lift weight	- Anatomical prints - Ultrasound biofeedback - A mirror - A light weight	- To contract the PFM and deep abdominal muscles when breathing out - To practice the PFM contraction before and during coughing and head lifting - To practice the knack when lifting a weight	- Ultrasound checking - A diary of the knack maneuver practice is requested
7	What are the best habits for the pelvic floor?	- Voiding ideal frequency - Infection prevention - Use of vaginal weight devices - Role of the pelvic floor in sexual intercourse	- Anatomical prints - A balloon - Vaginal weights	- To analyze the voiding frequency - To understand how the detrusor muscle and the PFM work - To list tips to avoid urine infections and improve sexual practice - To understand the use and guidelines for the use of vaginal devices	- To answer a short test about bladder function - To prepare your own “do’s and dont’s” as purposes to improve
8	What have I learned?	- All the contents of the program (session 1 to 7)	- Exploratory motivational interview - PIKQ - Real practical cases - Anatomical prints	- To fill in the PIKQ again - To detect the PFD symptoms, the risk factors, and the treatment options of each real practical case - To propose therapeutic resources to improve a PFD	- PIKQ & rubric of real practical cases are discussed

PFD: Pelvic Floor Disorders; PF: Pelvic Floor; PIKQ: Prolapse and Incontinence Knowledge Questionnaire; PFM: Pelvic Floor Muscles.

## Data Availability

The data presented in this study are available on request from the corresponding author. The data are not publicly available due to privacy or ethical restrictions.

## Supplementary Materials

The following are available online at https://www.mdpi.com/1660-4601/18/5/2377/s1, File S1: Spanish PIKQ; File S2: Real practical cases; File S3: Real Practical Case Rubric for Patients.
