# Supplementary material for: How Do We Assess Patient Skills in a Competence-Based Program? Assessment of Patient Competences Using the Spanish Version of the Prolapse and Incontinence Knowledge Questionnaire and Real Practical Cases in Women with Pelvic Floor Disorders
Source: Int J Environ Res Public Health. 2021 Mar 1;18(5):2377. doi: 10.3390/ijerph18052377 (PMC7967754; doi:10.3390/ijerph18052377)
Supplement: Supplementary file 1 [file ijerph-18-02377-s001.zip › Supplementary file S1_PIKQ.pdf]

ENCUESTA DE CONOCIMIENTOS SOBRE LA INCONTINENCIA Y EL PROLAPSO

Cuestionario a

A continuación, le presentamos una serie de preguntas sobre incontinencia urinaria (pérdida de orina o disfunción de la vejiga). Por favor, conteste en cada una de las preguntas si está usted de acuerdo, en desacuerdo o no lo sabe.

1. La incontinencia urinaria (pérdida de orina o disfunción de la vejiga) es más común en mujeres jóvenes que en mujeres mayores.

☐ De acuerdo ☐ Desacuerdo ☐ No lo sé

2. Las disfunciones de vejiga son más frecuentes en mujeres que en hombres.

☐ De acuerdo ☐ Desacuerdo ☐ No lo sé

3. No se puede hacer mucho para tratar las pérdidas de orina, a excepción del uso de compresas y pañales.

☐ De acuerdo ☐ Desacuerdo ☐ No lo sé

4. Para el tratamiento de la pérdida de orina **no** es importante realizar un diagnóstico previo del tipo de pérdida.

☐ De acuerdo ☐ Desacuerdo ☐ No lo sé

5. Las pérdidas de orina pueden deberse a múltiples causas.

☐ De acuerdo ☐ Desacuerdo ☐ No lo sé

6. Efectivamente, los ejercicios pueden ayudarnos a controlar la pérdida de orina.

☐ De acuerdo ☐ Desacuerdo ☐ No lo sé

7. Algunos medicamentos pueden causar la pérdida de orina.

☐ De acuerdo ☐ Desacuerdo ☐ No lo sé

8. Una vez que la persona comienza a perder orina, nunca vuelve a ser capaz de controlarla.

☐ De acuerdo ☐ Desacuerdo ☐ No lo sé

9. Para diagnosticar las pérdidas de orina los médicos pueden hacer test especiales según el tipo de disfunción de vejiga.

☐ De acuerdo ☐ Desacuerdo ☐ No lo sé

10. La cirugía es el único tratamiento para las pérdidas de orina.

☐ De acuerdo ☐ Desacuerdo ☐ No lo sé

11. Dar a luz muchas veces puede causar pérdidas de orina.

☐ De acuerdo ☐ Desacuerdo ☐ No lo sé

12. La mayoría de la gente que tiene pérdidas de orina puede curarse o mejorar con algún tipo de tratamiento.

☐ De acuerdo ☐ Desacuerdo ☐ No lo sé

Cuestionario b

A continuación, le presentamos una serie de preguntas sobre el prolapso de órganos pélvicos (que supone el descenso (prolapso) de la vagina, útero, vejiga o recto a través de la vagina). Por favor, conteste en cada una de las preguntas si está usted de acuerdo o en desacuerdo, o no lo sabe.

1. El prolapso de órganos pélvicos (descenso de vagina, útero, vejiga o recto) es más frecuente en mujeres jóvenes que en mujeres mayores.

   ☐ De acuerdo ☐ Desacuerdo ☐ No lo sé

2. Dar a luz muchas veces puede provocar prolapso de órganos pélvicos.

   ☐ De acuerdo ☐ Desacuerdo ☐ No lo sé

3. El prolapso de órganos pélvicos puede aparecer a cualquier edad.

   ☐ De acuerdo ☐ Desacuerdo ☐ No lo sé

4. Ciertos ejercicios pueden ayudar a detener o evitar que el prolapso de órganos pélvicos empeore.

   ☐ De acuerdo ☐ Desacuerdo ☐ No lo sé

5. Los síntomas del prolapso de órganos pélvicos pueden incluir pesadez y/o presión en la pelvis.

   ☐ De acuerdo ☐ Desacuerdo ☐ No lo sé

6. Examinar a la paciente es una buena manera para que un médico diagnostique un prolapso de órganos.

   ☐ De acuerdo ☐ Desacuerdo ☐ No lo sé

7. Una vez que la paciente tiene un prolapso de órganos pélvicos, **no** se puede hacer mucho para ayudarla.

☐ De acuerdo ☐ Desacuerdo ☐ No lo sé

8. Levantar peso a diario puede provocar un prolapso de órganos pélvicos.

☐ De acuerdo ☐ Desacuerdo ☐ No lo sé

9. La cirugía es un tipo de tratamiento para el prolapso de órganos pélvicos.

☐ De acuerdo ☐ Desacuerdo ☐ No lo sé

10. Los médicos pueden realizar una prueba en sangre para diagnosticar un prolapso de órganos pélvicos.

☐ De acuerdo ☐ Desacuerdo ☐ No lo sé

11. Los síntomas del prolapso de los órganos pélvicos pueden tratarse con un anillo de goma llamado pesario.

☐ De acuerdo ☐ Desacuerdo ☐ No lo sé

12. Las personas obesas tienen **menos** probabilidad de tener un prolapso de órganos pélvicos.

☐ De acuerdo ☐ Desacuerdo ☐ No lo sé
