# Supplementary material for: How Do We Assess Patient Skills in a Competence-Based Program? Assessment of Patient Competences Using the Spanish Version of the Prolapse and Incontinence Knowledge Questionnaire and Real Practical Cases in Women with Pelvic Floor Disorders
Source: Int J Environ Res Public Health. 2021 Mar 1;18(5):2377. doi: 10.3390/ijerph18052377 (PMC7967754; doi:10.3390/ijerph18052377)
Supplement: Supplementary file 1 [file ijerph-18-02377-s001.zip › Supplementary file S2_REAL PRACTICAL CASES.pdf]

File S2. Therapeutic patient education program for women with pelvic floor disorders. Competence assessment tool: **Real practical cases**.

<table>
<tr><td colspan="3">Case 1. María is 45 years old and has two children who were born by cesarean section. She is a very athletic woman, and she likes to go to the gym to practice Zumba and Pilates. María is very allergic to pollen and she has to go through a park with lots of trees and flowers to get to the gym. Spring has arrived and she does not stop sneezing every time she goes to the gym, with what she is feeling that she loses urine in these episodes. There are even days that she notices pee leaks in the gym. Also, the other day when she got home, she had a sudden urge to urinate, and she hardly reach the toilet. She is very worried and is thinking that the best solution will be to stop going to the gym. What is happening to María? What risk factors do you find for her possible pelvic floor dysfunction? Which healthcare professional should she go to? What would you recommend to her?</td></tr>
<tr><td>Competence: dimensions</td><td>Knowledge and behaviors</td><td>Achievement evaluation</td></tr>
<tr><td>Identify, analyze: identify and analyze the significant elements, summarize and integrate the different parts, organize the elements and the connection among them, deduce some ideas and/or results and provide some conclusions.</td><td>María has to
1. Identify that the present symptoms are warning signs and they have a solution, but they require consultation with professionals.
2. Know:
o Obstetric risk factors (births were by cesarea, which can prevent perineal trauma but do not prevent the pregnancy factor to weaken the pelvic floor and abdominal muscles that contributes to proper abdomino-pelvic synergistic function in regulating intra-abdominal pressure).
o Secondary risk factors related to sport and allergic condition (may contribute to repeated increases in pressure to pelvic floor).</td><td>1. Evaluation: knowledge test of stress urinary incontinence symptoms and strategies to improve them.
2. Rubric for practical real cases.</td></tr>
<tr><td>Decision making: apply systematic methods to take decisions, compile and analyze data to take the most suitable decision, show certainty, be consistent with the solution adopted, collaborate with other on taking decisions.</td><td>María has to
1. Assess their needs and objectives to take appropriate measures to achieve them.
2. Demand professional attention in the field of Physiotherapy and Medicine. For her urinary symptoms, she should go to a physiotherapist specializing in pelvic floor, who is the professional who can help her improve her symptomatology and prevent her from progressing. The physical therapist may inform you if it is appropriate for you to consult other professionals such as urologists or gynecologists. Collaborating with the physical therapist in the decision she could accept an assessment of her pelvic floor to detect a dysfunction in her musculature. If she conducts a treatment that</td><td>1. Behavior evaluation: Voiding dairy and therapeutic adherence to Knack.
2. Rubric for practical real cases.</td></tr>
</table>

	includes strengthening pelvic floor muscles and automating it during efforts, she can decrease or even eliminate your symptoms.<br>3. *Adopt new health behaviors:* María would not be suggested to stop going to the gym, but she could be able to modify her sports activities in favor of less impact-impact ones like elliptical or yoga. She can also try to avoid exposure to allergens and/or assess the need for medical consultation. As she has leaks in the gym and urgency, it would be advisable to assess her hidric and voiding habits in order to detect alterations in the frequency of urination and fluid intake.	
**Communicate needs:** intonation and volume, level of preparation of exposition, gestures and body language, clarity of exposure, ability to answer questions, speech clarity, structure, and sequence within the speech.	María has to<br>1. Employ an assertive communication, structured and clear to professionals, to express her needs and obtain the timely care.	Cognitive evaluation:<br>1. Argumentation paper on medicine and physiotherapy. Behavioral evaluation: supporting documents with obtaining treatment or follow-up.<br>2. Rubric for practical real cases.

Case 2. ***Three weeks ago, the gynecologist told Mercedes that she has a grade II rectocele and she should take care of herself. She has always been very constipated, and she is afraid to go to the toilet, preferring to hold the desire back to make no effort. What would we say to Mercedes?***		
**Competence: dimensions**	Knowledge and behaviors	Achievement evaluation
**Decision making:** apply systematic methods to take decisions, compile and analyze data to take the most suitable decision, show certainty, be consistent with the solution adopted, collaborate with other on taking decisions.	Mercedes has to<br>1. *Know* that delaying or avoiding defecation is not beneficial for improving constipation or protecting her pelvic floor. On the contrary, delaying the desire for defecation can harden stool and cause effort and defecatory time to be longer.<br>2. *In decision-making* to improve this aspect she will be recommended to try to plan and respect a defecation schedule, in addition to maintaining a diet rich in fruit and vegetables, an adequate intake of liquids and an active lifestyle (for example, go for a walk every day).	Evaluación:<br>1. Knowledge test on the physiology of defecation and defecatory diary<br>2. Rubric for practical real cases.

	Excessive thrust when defecating can contribuite Mercedes notice more her prolapse and evolve it over time to more advanced grades, requiring more invasive treatments. To facilitate defecation we will recommend a pelvic retroversion position with feet resting on a stool, so that these are supported and the knees are slightly above the height of the hips. The defecation push should be carried out on expiration and being aware that it relaxes the pelvic floor muscles. To protect the prolapse, she can hold the vulva when she has to push more. We recommend that after defecating she could do a series of pelvic floor muscles contractions, sitting or standing, always making sure that the prolapse is within the vagina.<br>3. Once she has known and evaluated these aspects she will have to *decide* on their integration into your daily life.	
**Problem solving:** define the problem, identify strategies, propose solutions/hypotheses, evaluate potential solutions, implement solutions, evaluate results.	Mercedes has to<br>1. *Define* her defecation problem.<br>2. Mercedes should *identify* as a strategy that going to a specialized physical therapist would be helpful to improve her process by improving the propioception of her pelvic floor muscles and improving her muscle qualities, both strengthening and learning how to relax them. Physical therapist could employ techniques such as therapeutic education, specific exercises of pelvic floor muscles, the use of intra-anal biofeedback and abdominal massages.<br>3. *Implement* this strategy and evaluate its results.	Cognitive evaluation:<br>1. Structured document identifying positive variables of change in her process.<br>2. Weekly diary completion with adopted strategies and perceived effects.<br>3. Rubric for practical real cases.

Case 3. ***Ana is 35 years old and she became a mother 6 months ago. A couple of months ago, she returned to normal sex life with her partner, but she notices that something in her body has changed. The penetration is bothering her, and that is something that has never happened to her. At first, she thought it would be from the episiotomy, but she has been checking the scar and when she touches it no longer hurts. In addition, she has paid more attention to her sensations, and she notices discomfort especially when penetration begins, and also at the bottom of the vagina, with a mixture between pressure and pain. What do you think is the matter? Can she do anything to improve these complaints?***		
**Competence: dimensions**	Knowledge and behaviors	Achievement evaluation
**Identify, analyze:** identify and analyze the significant elements,	Ana has to	Cognitive evaluation:

summarize and integrate the different parts, organize the elements and the connection among them, deduce some ideas and/or results and provide some conclusions.	1. *Know* the physiological changes after motherhood, the relationship with her sexual dysfunctions and the importance of knowing strategies in which her partner will be involved.<br>2. *Know* that pain during relationships may be due to some perineal injury (tear) or some contracture in the pelvic floor muscles, both from the deepest muscles, which can justify pain at the bottom of the vagina, as well as superficial muscles, which can justify pain at the begining of penetration. In this case, Ana may have vaginal dryness if she is breastfeeding her baby. This could explain the pain when starting penetration, which causes her not being able to actively relax the pelvic floor.	1. List of changes in pelvic floor after maternity and their relationship to sexual dysfunctions.<br>2. Rubric for practical real cases.
**Communicate needs:** intonation and volume, level of preparation of exposition, gestures and body language, clarity of exposure, ability to answer questions, speech clarity, structure, and sequence within the speech.	Ana has to<br>1. *Know* that structured communication with argumentation based on knowledge about your process is necessary.<br>2. *Develop* the ability to expose her feelings and needs to professionals and/or her partner.<br>3. *Express* to her partner assertively the need for changes in sexual intercourse that include sexual practices that are not necessarily based on vaginal penetration.<br>4. *Modify* her sexual behavior according to the expression performed.	Cognitive evaluation:<br>1. Simulation of reasoned exposure of arguments and feelings.<br>2. Rubric for practical real cases.
**Know how to manage:** identify the different resources, access resources, use resources efficiently, assess the suitability of resources based on the results of use.	Ana has to<br>1. *Identify* her problem and the resources she might have to improve it.<br>2. For the improvement in sex after motherhood it is necessary to know strategies for their management, know their advantages and disadvantages, recognize the involvement of her partner in this field and detect aspects that allow to evaluate the improvement of her process.<br>3. As strategies it would be proposed:<br>o To consult a physical therapist or her midwife or gynecologist to reject other possible sources of pain (infection, organ prolapse).<br>o A physical therapy treatment can reduce pain and improve the ability to relax the pelvic floor muscles.<br>o In addition, the use of aqueous-based lubricant during penetration would facilitate sexual intercourse in postpartum period.	Cognitive evaluation:<br>1. Elaboration of a scheme that relates problems/symptoms with strategies and evolution variables.<br>2. Rubric for practical real cases.
