# Supplementary material for: How Do We Assess Patient Skills in a Competence-Based Program? Assessment of Patient Competences Using the Spanish Version of the Prolapse and Incontinence Knowledge Questionnaire and Real Practical Cases in Women with Pelvic Floor Disorders
Source: Int J Environ Res Public Health. 2021 Mar 1;18(5):2377. doi: 10.3390/ijerph18052377 (PMC7967754; doi:10.3390/ijerph18052377)
Supplement: Supplementary file 1 [file ijerph-18-02377-s001.zip › Supplementary file S3_RUBRIC.pdf]

REAL PRACTICAL CASE RUBRIC FOR PATIENTS

for additional information contact maria.torres@uah.es

This rubric has been created by the Research Group "Physiotherapy in Women's Health" from Alcalá University and aims to be a learning aid for all those patients who, as part of their Physiotherapy treatment, participate in an educational program. Any questions about its use or the language used should be discussed with the program's physiotherapists.

Definition: The real practical case rubric for patients is an instrument that aims to promote learning and verify the acquisition of competences in patients who have participated in a therapeutic patient education program guided by the review of real practical cases. A practical case in this context is defined as a description of symptoms, signs, sensations, thoughts, beliefs or environmental factors that could occur in a patient when faced with a health disorder. These are, in short, typical examples that could resemble what a patient may experience at this time.

Conceptual framework of the rubric: The rubric is structured by specifying the competencies that are important for managing health processes and performance descriptors or criteria that help to verify what has been achieved so far in this regard. In this way, in "competition" it is known what aspect has been developed, and in "descriptors" it is known what has been achieved. For each competence, three levels of achievement are described: “You can keep improving”, “Well done” and “Gold medal” that place the patient at her learning level.

Glossary (some terms used in the rubric are clarified below):

- ✓ Therapeutic patient education program: coordinated set of educational activities led by health professionals for patients and their families. It contributes to the acquisition and maintenance of self-care skills, as well as the mobilization or acquisition of adaptation skills. It is implemented in a certain context and for a certain period in search of a new healthier health behavior.
- ✓ Competence: combination of knowledge, skills and attitudes that are used to improve health behaviors.
- ✓ Identify / analyze: it involves distinguishing important facts about my health, understanding how important they are, what they mean to me and the relationship between them.
- ✓ Communicate needs: once I identify my needs, I am able to transmit them to others, presenting my ideas with good intonation and volume, gestures and body language that improve communication, clarity in the presentation of my ideas and I have the ability to answer questions.
- ✓ Make decisions: to be a participant in my health I apply systematic methods to make decisions, I collect and analyze data to choose the most appropriate decision, I demonstrate security, I am consistent with the solution adopted and I collaborate with others to make decisions.
- ✓ Problem solving: when I face a health problem, I am able to define the problem, identify strategies, propose solutions / hypotheses, evaluate possible solutions, put them into practice and check their results.
- ✓ Know how to manage: to improve my healthy behaviors I can identify resources that I need, I know how to access them, I use them efficiently and I can evaluate the suitability of resources according to the results of their use.
- ✓ Need: it is everything related to my health that I identify or detect as essential or important to attend to, communicate, treat or improve it. To improve it, I can do it by myself and I can also express it to my environment or to a health professional so that they help me or understand me. For example: expressing my feelings, knowing common symptoms, knowing what to do in a moment regarding a problem, etc.
- ✓ Resource: help, means, instrument or procedure that is used to solve something. For example, a resource to improve my health can be Physiotherapy.

REAL PRACTICAL CASE RUBRIC FOR PATIENTS

When a level is indicated (GM, BH or KI) it means that everything described in the corresponding table has been achieved

Competence	Performance descriptors			
	Gold Medal (GM)	Well Done (WD)	You can keep improving (KI)	Level
Identify / Analyze	I identify and understand all the data and also acknowledge that I could improve my understanding of the case with other data, or that I do not even need more data to manage the health problem that arises.<br><br>I consistently relate the different data of the case and I can relate them to my health process.<br><br>The case data has meaning to me and its analysis helps me to understand my health process.	I clearly identify the important data of the case.<br><br>I understand the importance of most of the data and am able to make some relationships between them.<br><br>I coherently relate the case data, but I have difficulty relating them to my health process.<br><br>All case data have some meaning to me.	I identify with great difficulty the important data of the case.<br><br>I have difficulty analyzing / understanding the importance of data and making relationships between them.<br><br>I do not coherent relate the data to my health process.<br><br>The case data doesn't mean anything special to me.	
Communicate needs	I clearly identify the needs that should be expressed in the case.<br><br>I can state the needs neatly and clearly.<br><br>I know how to reinforce the needs I want to express with my tone and gestures.<br><br>I can answer any questions about the needs raised by the case.	I identify some needs that should be expressed in the case.<br><br>I can clearly and orderly state some needs.<br><br>I am clear about the tone and gestures that I would use to express my needs.<br><br>I can answer a question about the needs of the case.	I identify with some difficulty the needs that should be expressed in the case.<br><br>I do not know very well how to state the needs neatly and clearly.<br><br>I have difficulty adjusting the tone and gestures I use to express needs.<br><br>I have difficulty answering questions about the needs raised by the case.	

REAL PRACTICAL CASE RUBRIC FOR PATIENTS (continuation)

When a level is indicated (GM, BH or KI) it means that everything described in the corresponding table has been achieved.

Competence	Descriptores			
	Golden Medal (GM)	Well Done (WD)	You can keep improving (KI)	Level
Decision making	In an orderly manner, I am able to collect and analyze all the information necessary to make decisions.<br>I am sure of the decisions I would make.<br><br>I know clearly what decisions to make and how to carry them out.<br><br>I understand the need to collaborate with other people on decision making and I know how to do it.	I can collect and analyze some of the information necessary to make decisions.<br><br>I am quite certain about the decision I would make.<br><br>I know what decisions to make, but I don't know how to carry them out.<br><br>I can collaborate with other people on decision making.	I have great difficulty collecting and analyzing the information necessary to make decisions.<br><br>I would be undecided if I had to make decisions in this case.<br><br>I have difficulties knowing what decisions to make and how to carry them out.<br><br>I have difficulty knowing how to collaborate with other people on making my decisions.	
Problem solving	I define the stated problem clearly.<br><br>I can identify strategies to solve problems and propose solutions to put into practice.<br><br>I have criteria to evaluate the different results of the proposed solutions.	I have difficulty defining the problem posed.<br><br>I can identify some strategies to solve the problems, but I have difficulty proposing solutions to implement.<br><br>I can evaluate some results of the proposed solutions.	I have difficulty defining the problem posed.<br><br>I have difficulty identifying strategies and proposing solutions to put into practice.<br><br>I have difficulty knowing how to evaluate the results of the proposed solutions.	
Management skills	I clearly identify all the necessary resources.<br><br>I know how and where to look and how to access all resources.<br><br>I know how to use resources and evaluate its suitability based on the results of its use.	I can identify most of the resources needed.<br><br>I know how and where to search and how to access some of the resources.<br><br>I know how to use the resources, although it would be difficult for me to assess their suitability based on the results of their use.	I am having difficulty identifying necessary resources.<br><br>I am having difficulty knowing how to search and access resources.<br><br>I am having difficulty knowing how to use the resources and assessing their suitability based on the results of their use.	
